# Description of a new species of *Membranobalanus* (Crustacea, Cirripedia) from southern Australia

**DOI:** 10.3897/zookeys.873.35421

**Published:** 2019-08-29

**Authors:** Andrew M. Hosie, Jane Fromont, Kylie Munyard, Diana S. Jones

**Affiliations:** 1 Aquatic Zoology, Western Australian Museum, 49 Kew St, Welshpool 6106 WA, Australia Curtin University Bentley Australia; 2 School of Pharmacy & Biomedical Sciences, Curtin Health Innovation Research Institute, Curtin University, Bentley, 6102 WA, Australia Western Australian Museum Welshpool Australia

**Keywords:** Archaeobalanidae, bioeroder, commensal, Clionaidae, computed tomography, Demospongiae, specificity, *Spheciospongia purpurea*, symbiosis, temperate reef.

## Abstract

A new species of sponge-inhabiting barnacle, *Membranobalanus porphyrophilus***sp. nov.**, is described herein. This species can be distinguished from all other congeners by a combination of characters, in particular by the shapes of the tergum and scutum and the armament of the cirri. COI sequence data from the type specimens have been lodged with GenBank and a morphological key to the species of *Membranobalanus* is provided to aid future research. The host of the new species is the southern Australian endemic demosponge *Spheciospongia purpurea*. The new species of barnacle is thought to be host species specific.

## Introduction

Barnacles of the genus *Membranobalanus* Hoek, 1913 are obligate symbionts of sponges. While the identity of sponge hosts for most sponge-dwelling barnacles have been poorly documented, this is not the case for *Membranobalanus*. All species have been found embedded only in the genera *Cliona* Grant, 1826 and *Spheciospongia* Marshall, 1892 in the family Clionaidae D’Orbigny, 1851 (van Syoc 1988; van Syoc et al. 2015), with the exception of *M. longirostrum* (Hoek, 1913), which has been additionally reported from the genus *Suberites* Nardo, 1833 in the family SuberitidaeSchmidt 1870 (Ilan et al. 1999; Wibowo et al. 2011; Sulistiono et al. 2014) and *M. orcuttiformis* (Kolosváry, 1941) where no host has been reported.

The Clionaidae (Demospongiae, Clionaida) is a group of sponges well known for bioeroding calcareous structures such as mollusc shells and scleractinian coral skeletons (Rutzler 1975). Sponges in the genus *Spheciospongia* excavate limestone substrates in early life history stages and can become massive sponges with age (Vicente et al. 1991). The barnacles are found embedded within the host, the tissues of which are in direct contact with the calcareous parietes of the barnacles, seemingly undeterred by the potential bioeroding effects of the sponge.

This study describes a new species *Membranobalanus* collected as part of a broader study on sponge-inhabiting barnacles in Australian waters. The host species, *Spheciospongia purpurea* (Lamarck, 1815), is endemic to southern Australia and easily recognised due to its vibrant purple colouration, which it retains even in ethanol or a dry state. The dense royal purple pigment reported in this species and other species of *Cliona* and *Spheciospongia* is a porphyrin, specifically spongioporphyrin (Bergquist 1978).

## Methods

Prior to dissection, the designated holotype was scanned via μCT using a Zeiss Versa XRM-520 X-ray microscope at the Centre for Microscopy, Characterisation and Analysis at the University of Western Australia. Processing of the resulting data followed the methods described in Semple et al. (2019) using the software packages Drishti 2.6.4 (Limaye 2012), Meshlab 2016 (Cignoni et al. 2008), and Adobe Acrobat Pro X.

For direct morphological examination of barnacle shell plates and arthropodal characters, the body and associated soft tissues were removed from the shell. The remnants of the barnacle tissue and host sponge on the surfaces of the parietes, scutum and tergum were removed using forceps. The shell was then immersed in 2% bleach for ~2 h to completely digest the organic tissue and subsequently rinsed in purified water. Any remaining debris or contaminants were then removed by cleaning in an ultrasonic cleaner for less than 20 s for shell plates and 5 s for arthropodal parts. The specimens were examined under a Leica M205 C (Leica, Germany) stereomicroscope and digital photographs produced with a Leica DMC4500. For scanning electron microscopy, specimens were first dehydrated in an ethanol series (70%, 80%, 90%, 100%, 5 minutes each) then transferred to hexamethyldisilazane for 10 min. Excess liquid was then removed with an eye-dropper and specimens were left to dry in a fume hood for 30 min. The dissected specimens were mounted on stubs, sputter coated with gold, and observed using a Hitachi TM3030 tabletop SEM. All images were processed using Adobe Photoshop CS3.

Adductor or depressor muscle tissues were subsampled from specimens, and genomic DNA was extracted using either a Bioline Isolate II or Qiagen DNeasy extraction kit following the manufacturers’ instructions. Partial fragments of the cytochrome c oxidase I gene were amplified using the primers dgLCO1490 5'-GGTCAACAAATCATAAAGAYATYGG-3' and dgHCO2198 5'-GGTCAACAAATCATAAAGAYATYGG-3' (Meyer et al. 2003) in a 25 µL reaction volume consisting of 1 unit MyTaq DNA polymerase, 1× MyTaq PCR buffer, 0.5 µL of each primer, and 2 µL template. The following polymerase chain reaction conditions were used: 2 min at 95 °C for initial denaturing, then 35 cycles of 30 s at 95 °C, 30 s at 46 °C, 45 s at 72 °C, and a final extension for 7 min at 72 °C. The resulting amplicons were sequenced by the Australian Genome Research Facility, Perth, using the same primers. The sequences were assembled and trimmed using Geneious Prime and submitted to GenBank (Table 1; https://www.ncbi.nlm.nih.gov/genbank/).

**Table 1.** Accession details for COI sequences of *Membranobalanus porphyrophilus* sp. nov. deposited with GenBank.

Specimen catalogue	GenBank #
WAM C66803	MK900684
WAM C71853	MK789771
WAM C71881	MK789772

Specimens of both barnacles and sponges are housed at the Western Australian Museum, Perth (WAM) and South Australian Museum, Adelaide (SAMA).

## Systematics

### Suborder Balanomorpha Pilsbry, 1916

#### Superfamily Balanoidea Leach, 1817

##### Family Archaeobalanidae Newman & Ross, 1976

###### Membranobalanus

Taxon classificationAnimaliaSessiliaArchaeobalanidae

Hoek, 1913

CBA07DDC8B85516BBC8CA34CC387C3D3

####### Type species.

*Balanus declivis* Darwin, 1854: 275, pl. 7 fig. 4a–d; by subsequent designation (Pilsbry 1916: 229).

####### Species composition.

*M. brachialis* (Rosell, 1972); *M. costatus* Zullo & Standing, 1983; *M. cuneiformis* (Hiro, 1936); *M. declivis* (Darwin, 1854); *M. koreanus* Kim & Kim, 1983; *M. longirostrum* (Hoek, 1913); *M. nebrias* (Zullo & Beach, 1973); *M. orcutti* (Pilsbry, 1907); *M. porphyrophilus* sp. nov.; *M. robinae* Van Syoc, 1988.

####### Nomen dubium.

*M. orcuttiformis* (Kolosváry, 1941).

####### Diagnosis.

Parietes solid, unornamented, weakly articulated, basis membranous. Rostrum scoop or boat-shaped, often elongate relative to other parietes. Tergum with spur furrow open. Cirrus IV with erect spines, with or without recurved teeth on anterior ramus.

####### Remarks.

With the addition of the below described species, there are now 10 species included within *Membranobalanus*. Utinomi (1968) synonymised the taxa Balanus (Membranobalanus) longirostrum var. krusadaiensis Daniel, 1955, B. (M.) basicupula Suhaimi, 1966, and B. (M.) roonwali Prem-Kumar & Daniel, 1968 under *M. longirostrum*, proposing that the differences observed are within the bounds of intraspecific variability. Recently, van Syoc et al. (2015) transferred *Acasta acuta* (Kolbasov, 1993) out of *Membranobalanus* based primarily on the presence of calcareous spines on the parietal wall, a character no other *Membranobalanus* possess and in reference to a cladistic analysis in an unpublished thesis. The fact that *A. acuta* is found in sponges of the family Petrosiidae (order Haplosclerida), not the Clionaidae as reported for the remaining members of the genus, separates this species ecologically from *Membranobalanus*, was used as further justification. The general appearance, membranous basis and elongated rostrum of *A. acuta* are typical features of *Membranobalanus*, however. While we treat this reassignment with caution, we have no evidence with which to dispute it.

Kolosváry (1941) described Balanus (M.) orcuttiformis based on the parietes of a single empty specimen. The locality details of the specimen are vague, only given as “India Orient.”, but presumably meaning eastern India. No detail regarding a host was given. The description is very brief, giving very few clues to the identification of this species, and the only illustration of the specimen, in lateral view, could belong to a number of genera, but not *Membranobalanus* as currently defined. Most notable is the absence of any elongation of the rostrum and the largely horizontal basal rim of the parietal wall gives the appearance that it was attached to, rather than embedded within, a substrate. Additionally, the exceptionally broad alae and absent radii are reminiscent of the Pachylasmatoidea, which possess solid parietes and often have a membranous base (see Jones 2000). *Membranobalanus orcuttiformis* has not been recorded since its description and unfortunately the specimens are missing from the Museo di Storia Naturale dell’Università di Firenze, Italy and thus cannot be reexamined (Innocenti 2006). For these reasons, this species is considered herein a *nomen dubium* and has been excluded from the key below.

The remaining *Membranobalanus* species can be separated into two morphological lineages, approximating an American centred group and an Indo-West Pacific group. The former have recurved teeth, similar to those present in some members of the Acastinae, as well as erect spines on cirrus IV, smooth growth lines on the scutum and the articular ridge and groove of the scutum is prominent, extending well beyond the articular margin, with a correspondingly wide articular groove on the tergum. The latter group bears only the erect spines on cirrus IV, finely striated growth lines, and has relatively weak articular structures on the opercular plates. From a biogeographic perspective one species disrupts this pattern: *Membranobalanus koreanus* from the waters around the Korean Peninsula. As described and figured by Kim and Kim (1983), *M. koreanus* has recurved teeth and a large articular ridge on the scutum. The records of *M. orcutti* by Barnard (1924) and Rosell (1973, 1975) from South Africa and the Sulu Archipelago, respectively, were considered suspect by Zullo and Beach (1973) and Van Syoc and Winther (1999). The Sulu Archipelago specimens lack the recurved teeth on cirrus IV of the specimens described from Catalina Island by Zullo and Beach (1973), and while Barnard’s description specifically mentions recurved teeth on cirrus IV, most of his description is deferred back to either Pilsbry’s (1907, 1916) descriptions of *M. orcutti* or Hoek’s (1913) description of *M. longirostrum.* Of particular note in Barnard’s description is that the scutum has an external, setose membrane, a character seen in some species of the Acastinae, but not *Membranobalanus*. Both reports should be considered *species inquirenda*, but potentially represent previously undescribed species.

###### Membranobalanus porphyrophilus

Taxon classificationAnimaliaSessiliaArchaeobalanidae

Hosie & Jones sp. nov.

44C68B7595C95AE795395E0E5282D178

http://zoobank.org/170E814F-E3ED-4120-8622-EC4230353C65

**Figure 1.**
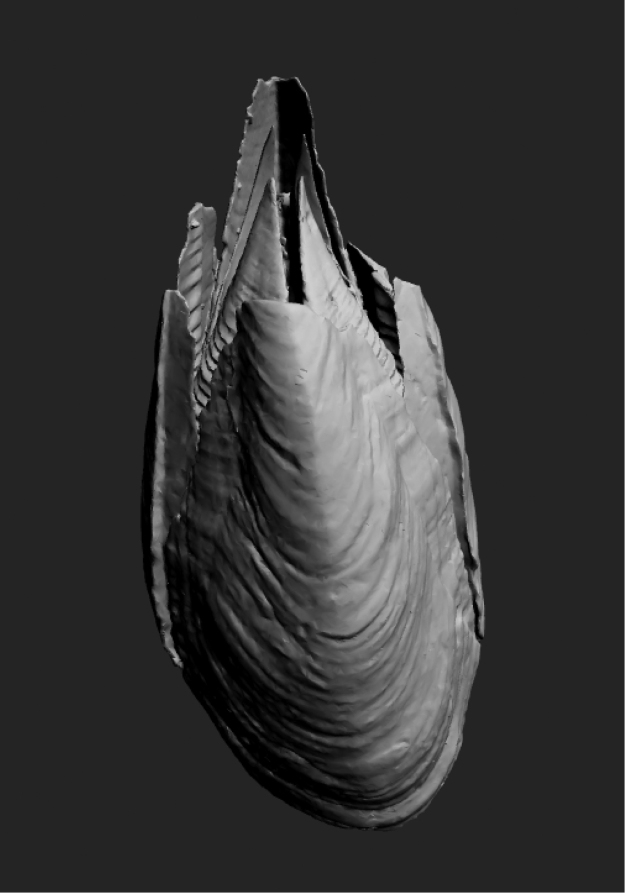
Interactive 3D, μCT derived volume reconstruction of *Membranobalanus porphyrophilus* sp. nov. holotype (WAM C66803). Only the well-calcified plates are illustrated as scanning limitations prevented the softer prosoma from being differentiated. Note: To enable the interactive function of this figure, open the PDF in Adobe Reader program or web plug-in.

**Figure 2.**
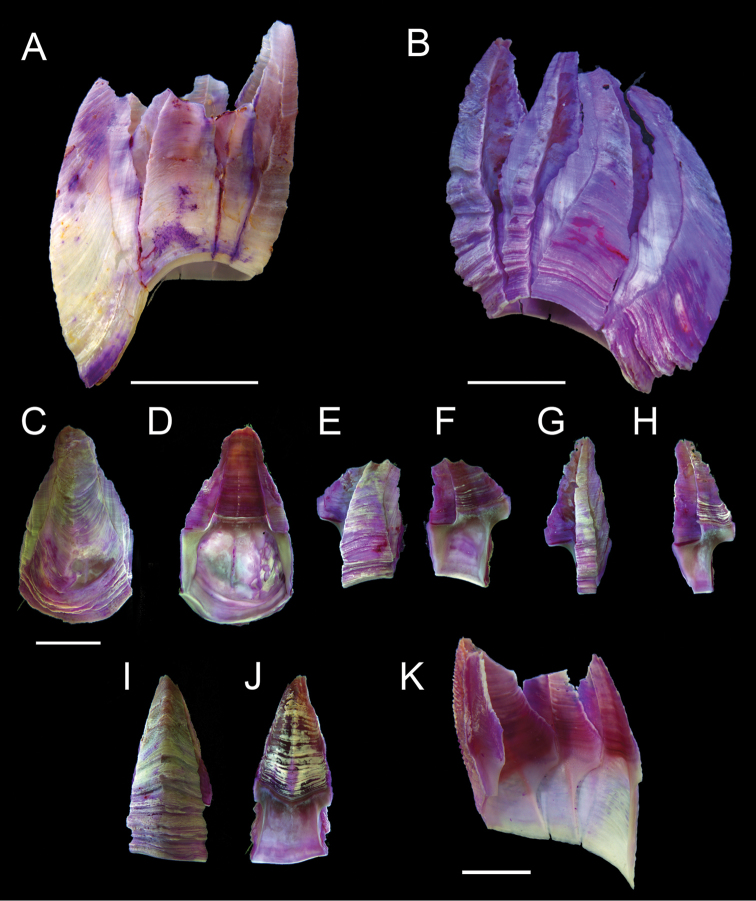
*Membranobalanus porphyrophilus* sp. nov. parietes **A** holotype (WAM C66803) **B–J** paratype (SAMA C12708) **K** paratype (SAMA C12706). **A** Whole shell lateral view **B** whole shell lateral view **C, D** rostrum, external and internal view **E, F** left lateral plate, external, and internal view **G, H** left carinolateral, external and internal view **I, J** carina external and internal view **K** internal view of articulated right carina, carinolateral and lateral plates. Scale bars: 5 mm (**A–J**); 2 mm (**K**).

**Figure 3.**
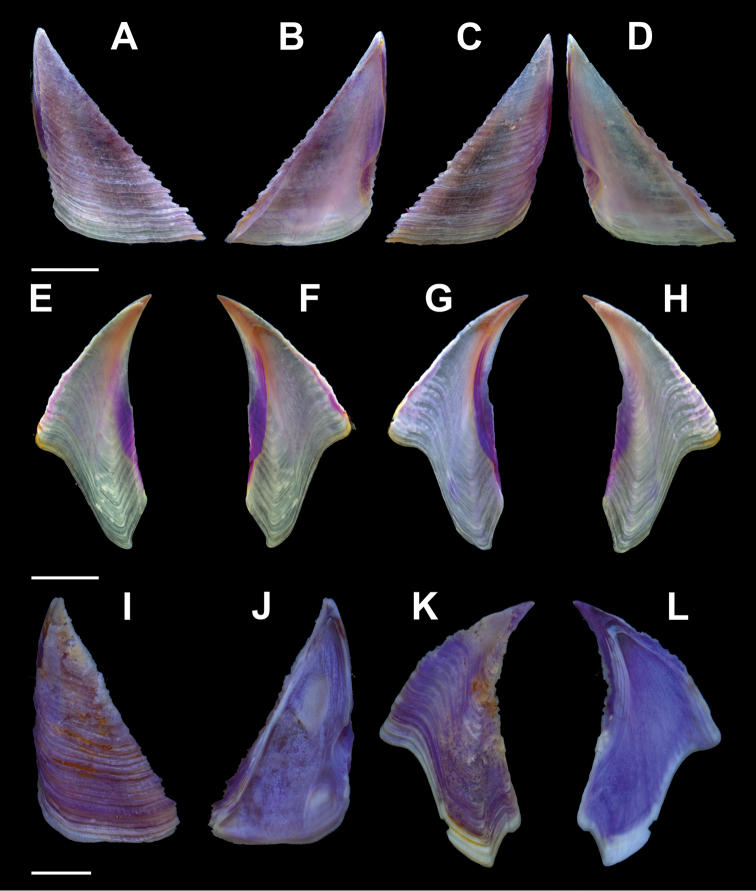
*Membranobalanus porphyrophilus* sp. nov. opercular plates **A–H** holotype (WAM C66803) **I–L** paratype (SAMA C12708). **A** Left scutum, external view **B** left scutum, internal view **C** right scutum, external view **D** right scutum, internal view **E** left tergum, external view **F** left tergum, internal view **G** right tergum, external view **H** right tergum, internal view **I** left scutum, external view **J** left scutum, internal view **K** left tergum, external view **L** left tergum, internal view. Scale bars: 2 mm.

**Figure 4.**
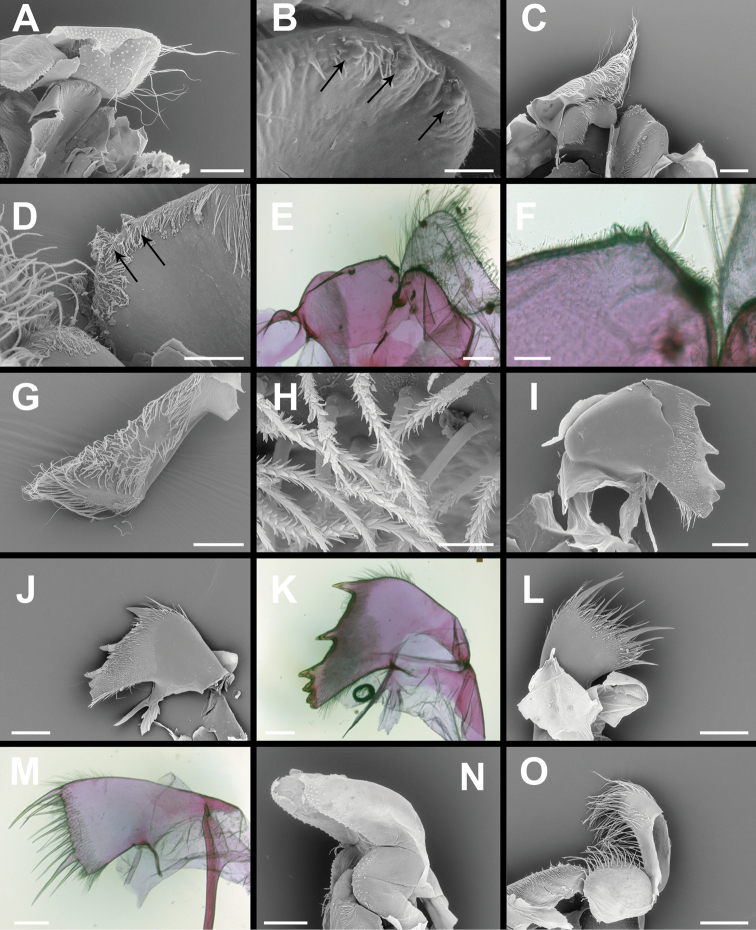
*Membranobalanus porphyrophilus* sp. nov. mouthparts **A, B, G, H, I, L, N** holotype (WAM C66803) **C, D, J, O** paratype (SAMA C12708) **E, F, K, M** paratype (SAMA C12706). **A, C** Labrum and right mandiblular palp (left removed, damaged) **B, D** close up of A and D, arrows indicate teeth on labrum **E** left mandibular palp **F** detail of serrulate setae on mandibular palp ventral face **G–I** mandibles **J** maxillule **K, L** maxilla. Note: G, H, L damaged, setae lost during sonication in A & K. Scale bars: 200 μm (**A, C, E, G, I–O**); 40 μm (**B, F**); 100 μm (**D**); 50 μm (**H**).

**Figure 5.**
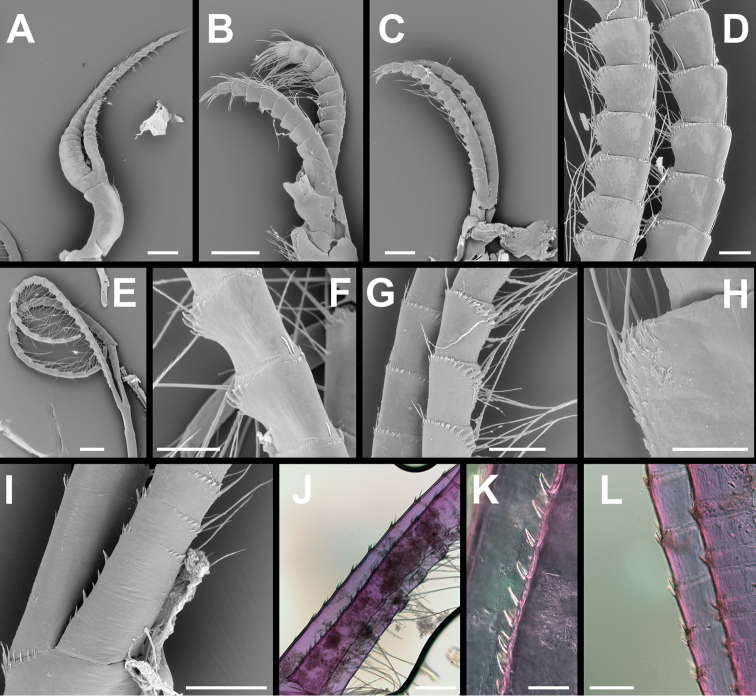
*Membranobalanus porphyrophilus* sp. nov. cirri **A–F** holotype (WAM C66803) **G–I** paratype (SAMA C12706) **J–L** paratype (SAMA C12708). **A–C** Right cirrus I–III lateral view **D** right cirrus III intermediate segments **E** right cirrus IV lateral view **F** same, anterior ramus intermediate segments **G** left cirrus IV anterior ramus intermediate segments **H** right cirrus IV anterodistal angle of distal segment of pedical showing ctenoid scales **I, J** Ieft cirrus IV basal segments of rami showing posterior spines **K** close up of spines on posterior margin of J **L** close up of spines and posterodistal spines basal segments of J. Note: B, C, E damaged. Scale bars: 400 μm (**A–C, E**); 100 μm (**D, F, L**); 150 μm (**G, I**); 50 μm (**H, K**); 200 μm (**J**).

**Figure 6.**
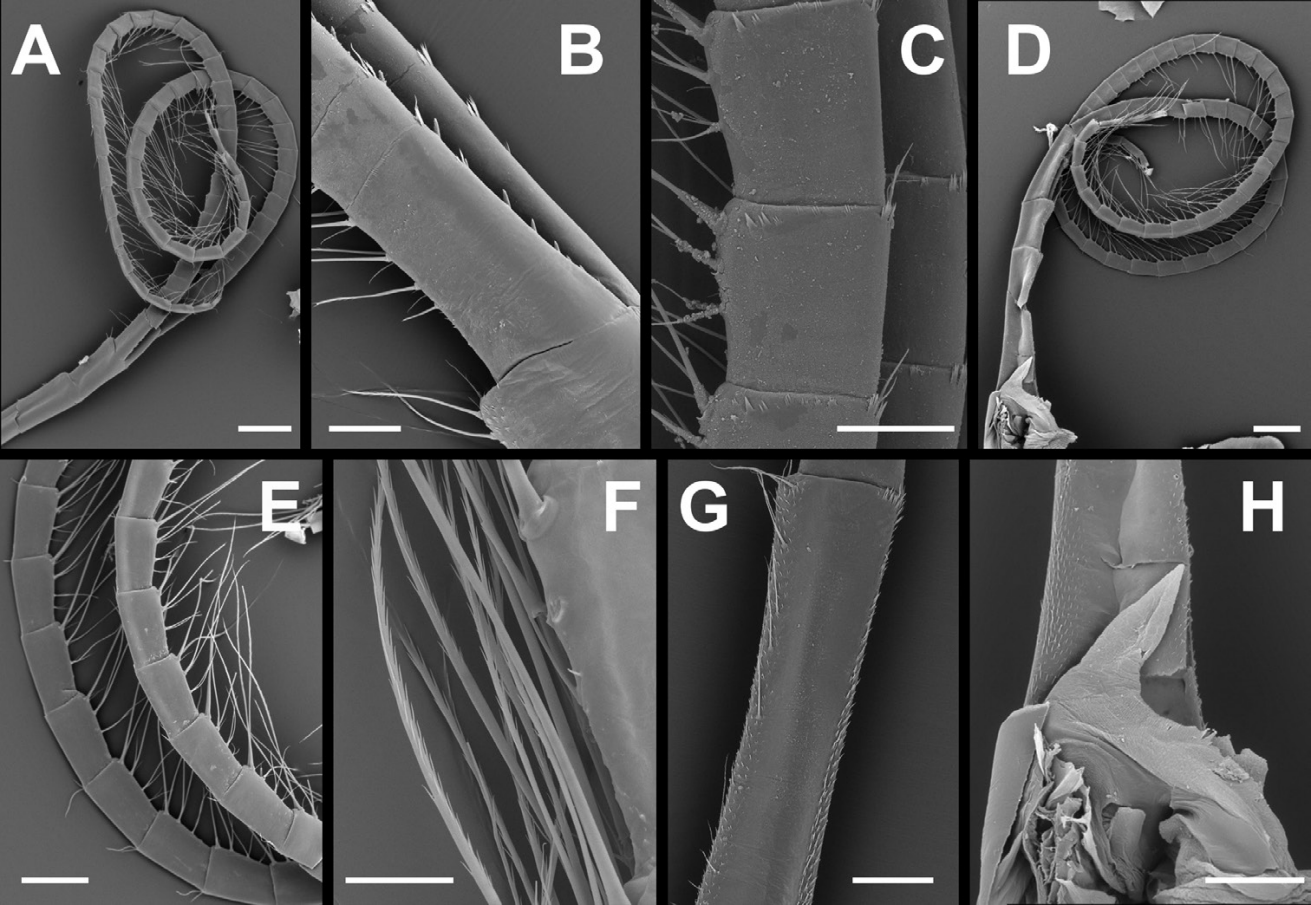
*Membranobalanus porphyrophilus* sp. nov. cirri **A, B, D, E, H** holotype (WAM C66803) **C, F, G** paratype (SAMA C12706). **A** Right cirrus V lateral view **B** basal segments of cirrus V rami **C** intermediate segments of cirrus V anterior ramus **D** right cirrus VI mesial view (damaged) **E** same, intermediate segments **F** example of long serrulate setae on cirrus VI **G** basal segment of pedicel of right cirrus VI, mesial view showing denticles on anterior and posterior margin **H** basidorsal point of penis. Scale bars: 400 μm (**A, D**); 100 μm (**B, C)**; 50 μm (**F**); 150 μm (**G, H**); 80 μm (**E**).

Figures 1 , 2 , 3 , 4 , 5 , 6

####### Material examined.

**Holotype.** AUSTRALIA • WAM C66803, 1 hermaphrodite; 9 mm rostro-carinal diameter; Western Australia SE of Rottnest Island, Wallace Island, The Count; 32°0.89'S, 115°33.53'E; 12 m; coll. A.M. Hosie; 23 Feb 2017; host: WAM Z86929, *Spheciospongia purpurea*.

**Paratypes.** AUSTRALIA • WAM C71852, 1 hermaphrodite; 8 mm rostro-carinal diameter; empty shell; same as data as for holotype. • WAM C71853, 1 hermaphrodite; same as data as for holotype. • WAM C71881, 1 hermaphrodite; same as data as for holotype. • SAMA C12706, 1 hermaphrodite; South Australia, Kangaroo Island, off Second Gully between Western River Cove and Snug Cove; 32 m; coll. J. Thiselton; 19 Nov 2002; host: SAMA S2910, *S. purpurea*. • SAMA C12707, 1 hermaphrodite; same data as for previous. • SAMA C12708, 1 hermaphrodite; 14 mm rostro-carinal diameter; same data as for previous.

####### Diagnosis.

Shell wall robust, cylindrical, growth ridges weak; orifice toothed, large; rostrum basal margin broadly rounded, extending below basal plane of remaining parietes. Tergum narrow, beaked, spur narrow, separated from basiscutal angle by half its own width; scutum with faint, external longitudinal striations; basitergal angle broadly rounded. Cirri III and IV with row of strong, erect spines on anterodistal margin of anterior ramus; cirrus IV pedicel without erect spines; cirri IV and V with row of stout spines on posterior margin of anterior ramus basal segment.

####### Description.

All shell plates, prosoma, and internal organs stained purple *in vivo*, otherwise white. Shell walls (Figs 1, 2) parallel, except bowed rostrum; parietes externally with horizontal growth lines raised, ridge-like, giving shell a roughened appearance; radii prominent, summits oblique, sutural edges roughened, alae wide, summits oblique. Internally parietes smooth, sheath occupying approximately half of shell height, with horizontal growth lines, basal margin adpressed. Carina approximately as wide as lateral plate, carinolateral narrowest plate, approximately one-third width of lateral plate. Rostrum elongate, extending below basal margin of other parietes, almost twice length and twice width of lateral plate, basal margin broadly rounded below basal margin of latera. Basal margins of carina and carinolatera more or less perpendicular to shell vertical axis, latera basal margins curving basally to form contiguous rim connecting latera with rostrum. Basis membranous, follows contour of basal rim, not depending below parietes.

Scutum (Fig. 3A–D, I, J) triangular, height 1.3–1.8 times width, growth lines prominent with faint longitudinal striations; basal and tergal margins separated by broadly rounded basitergal angle. Internal surface, slightly ridged apically near tergal margin, otherwise smooth; adductor muscle pit distinct; lateral depressor muscle pit distinct, without depressor muscle crests, extending one-third distance to beginning of articular ridge bounded by low, rounded adductor muscle ridge. Articular groove deep, narrow; articular ridge barely projecting beyond articular margin, basal margin curved.

Tergum (Fig. 3E–H, K, L) narrow, height more than twice width, articular and carinal margins arcuate with beaked apex; external growth lines conspicuous, but less raised than those of scutum; spur furrow indicated by shift in growth lines and slight depression; basiscutal angle sloping into spur; spur narrow, separated from basiscutal angle by half its width, basally truncate. Internally smooth, depressor muscle crests weak (may be absent in small specimens), articular groove wide, open, shallow; articular ridge low, becoming confluent with scutal margin basally.

Labrum (Fig. 4A–F) bilobed, with deep medial notch bounded by rounded crests, each with two or three marginal teeth and numerous fine setae.

Mandibular palp (Fig. 4A, C, E, G, H) rhomboid, apex obliquely truncate, anterior margin concave, posterior margin straight; setae (Fig. 4F) heavily serrulate, becoming longer and denser distally.

Mandible (Fig. 4I–K) with four distinct teeth, 2^nd^ and 3^rd^ tooth bifid, 4^th^ tooth much smaller than preceding three, 5^th^ tooth obsolescent, confluent with molariform inferior angle; short setae covering inner and outer faces, longer fine setae on inferior and superior margins.

Maxillule (Fig. 4l, M) with 10 robust setae on cutting margin, first, second, and ninth longer and more robust than remaining setae, inferior angle with several short robust setae. Cutting margin straight, with very slight notch below second seta. Dense, short setae regularly spaced on inner and outer faces, longer fine setae on inferior and superior margin

Maxilla (Fig. 4N, O) bilobed, basal lobe ovate, serrulate setae arranged on anterior margin; distal lobe elongate, serrulate setae on anterior margin becoming more dense at apex, longer than those on basal lobe.

Cirrus I (Fig. 5A) with unequal rami, anterior ramus twice length of posterior ramus, both rami bearing serrulate and simple setae. Posterior ramus segments with protuberant anterior margins, more densely setose than anterior ramus, arranged in tufts on anterior margins.

Cirrus II (Fig. 5B) shorter than other cirri, rami subequal; anterior margins of both rami slightly protuberant with serrulate and simple setae.

Cirrus III (Fig. 5C, D) pedicel with plumose setae on anterior and posterior margins of both segments; rami equal; anterior ramus with erect spines and ctenoid scales on anterodistal portion of medial segments, tuft of long serrulate setae on rounded anterior margin up to approximately three times as long as segment, tuft of serrulate setae as long as segment at posterodistal angle.

Cirrus IV (Fig. 5E–L) pedicel without erect teeth on anterodistal margins, pedicel with numerous small denticles on posterior and anterior margins of mesial face, tuft of short setae at posterodistal angle, small ctenoid scale-like denticles at anterodistal margin. Rami subequal in length; basal-most segment with up to 13 stout spines along posterior margin (holotype with six), basal segments with one or two stout spines at posterodistal angle. Anterior ramus intermediate segments with row of erect spines on anterodistal portion of all but most distal segments, much reduced in posterior ramus, without recurved teeth on anterior faces, posterodistal angles with tuft of setae.

Cirrus V (Fig. 6A–C) characters intermediate between cirri IV and VI; pedicel with numerous small denticles on posterior and anterior margins of mesial face, tuft of short setae at posterodistal angle, small ctenoid scale-like denticles on anterodistal margin. Rami equal, segments becoming elongate distally from half as long as wide to three times longer than wide, both rami with four pairs of serrulate setae on anterior margin, distal most pair longest, approximately 3 times length of segment; tuft of simple setae at anterodistal angle, setae up to half length of segment; erect teeth on anterior margins of intermediate segments less pronounced than those on cirrus IV; stout spines on posterior margins of rami similar to cirrus IV.

Cirrus VI (Fig. 6D–G) rami equal, pedicel with numerous small denticles on posteromesial face, tuft of simple setae at posterodistal angle; intermediate segments of both rami becoming elongate distally from 1.5 times wider than long to three times longer than wide, both rami with four pairs of serrulate setae on anterior margin, distal most pair longest, approximately three times the length of segment, basal pair 0.3 times length of segment; tuft of simple setae at anterodistal angle, setae up to 0.5 length of segment; anterior margins of all segments without erect teeth, posterior margins without stout spines.

Cirral segment counts as follows (anterior ramus, posterior ramus):

**Table:** 

	**Cirrus**	**I**	**II**	**III**	**IV**	**V**	**VI**
WAM C66803	L	21, 9	9, 11	17, 13	27, 30	34, 32	33, 33
	R	20, 9	11, 9	17, 14	30, 30	32, 34	36, 34

Penis longer than CVI, annulated, sparsely setose along length; basidorsal point (Fig. 6H) prominent, triangular, height twice width.

####### Distribution.

Southern Australia, from Perth to Adelaide.

####### Etymology.

From Greek *porphyra*, purple and *philos*, indicating a love of; gender masculine. In reference to the vibrant purple colour of the only known host.

####### Remarks.

*Membranobalanus porphyrophilus* sp. nov. is most readily distinguished from its congeners by the narrow, beaked tergum. The absence of recurved teeth on cirrus IV, the finely striated scutal growth lines, and the weak articular structure of the opercular plates further separates the newly described species from the predominantly American group of species, viz. *M. costatus*, *M. declivis*, *M. koreanus*, *M. nebrias*, *M. orcutti*, and *M. robinae*. The remaining species in the genus, *M. brachialis*, *M. cuneiformis*, and *M. longirostrum* all bear prominent, erect spines on the pedicel of cirrus IV and lack the stout spines on the posterior margins of the rami of cirri IV–VI. This is only the second member of the genus reported from Australian waters. The other, *M. cuneiformis*, is known from near Darwin and was reported by Hiro (1936) as commensal with an unidentified species of *Cliona* that was bioeroding the shell of *Pinctada maxima* (Jameson, 1901). Externally, *M. cuneiformis* can be separated from *M. porphyrophilus* by the conical shape of the shell, resulting in a small orifice and absent radii.

## Discussion

Only a single sequence, identified as *M. longirostrum* (GenBank accession #KC138493; Chen et al. 2013), represents the genus *Membranobalanus* on GenBank, and the sequences generated herein are less than 85% similar, indicating a rather distant relationship with *M. porphyrophilus* sp. nov.

The degree of elongation and curvature of the rostrum is variable in *M. porphyrophilus* sp. nov. and does not appear to be directly related to increasing size, as the rostrum of both the larger and smaller specimens can be relatively short. The development of the rostrum will be in large part an adaptation to prevent being overgrown by the host, and it is likely that the aspects of the rostral form will be determined by the placement of the barnacle relative to the direction of growth of the host.

While *Spheciospongia* are bioeroders generally only in early life history stages, the bioeroding capacity does appear to have impacted on the barnacle shells. The older parts of the shell plates are often pitted and scarred, indicative of the potential bioeroding effects of the sponge. The shell plates, exoskeleton, and tissues are brightly stained by the purple pigments of the host sponge (persistent even in ethanol), and are the most obvious impact of the sponge on the barnacle.

*Spheciospongia purpurea* has had a complex taxonomic history and at one stage many species were listed in synonymy and thus the species was considered to be widely distributed (see Vosmaer 1911: 6 for list of synonyms). Topsent (1918) brought many of these species back out of synonymy and *S. purpurea* is now considered to be endemic to southern Australia (Atlas of Living Australia 2019). In Hoek’s (1913) report on the cirripedes collected during the *Siboga* Expedition he listed *S. purpurea* as the host sponge in his description of *M. longirostrum* collected from the seas around Indonesia, far outside the known range of *S. purpurea*. Hoek’s record is, thus, considered to be a misidentification, most likely based on the identifications in Vosmaer’s (1911) account of the sponges from the same expedition. It is unclear if the records of *S. purpurea* as the host by Rosell (1972) from the Philippines and by Utinomi (1968) from Japan are based on their material or simply repeated from Hoek’s report, but here they are considered to be errors for the same reason.

No other barnacle species were found inhabiting any of the 15 *S. purpurea* specimens examined during the course of this study. Over 200 morphospecies of sponge have been found to host barnacles in Australian waters (Hosie and Fromont unpublished data), including five other species of *Spheciospongia*, and *Membranobalanus porphyrophilus* sp. nov. has been found inhabiting only *S. purpurea*, making it likely to be restricted to this species and therefore also an Australian endemic.

Host specificity of *Membranobalanus* was first discussed by van Syoc (1988), and the newly described species supports the hypothesis that they are restricted to inhabiting species of the Clionaidae. The exception to this is *M. longirostrum*, which has also been reported inhabiting species of the Suberitidae in the Red Sea (Ilan et al. 1999) and Indonesia (Wibowo et al. 2011; Sulistiono et al. 2014). The potential for the misidentification of host species by Ilan et al. (1999) was raised by van Syoc (2015), as the genus *Suberites* is morphologically similar to *Spheciospongia.* This similarity has led to species of both genera being confounded; as an example, *Suberites wilsoni* Carter, 1885 is considered a subjective synonym of *S. purpurea*. The existing records of *Membranobalanus* embedded in *Suberites*, as well as any future collections, need to be examined by sponge specialists to determine if they have been correctly assigned.

### Key to the genus *Membranobalanus*

**Table:** 

1	Cirrus IV anterior ramus bearing recurved teeth and erect spines	**2**
–	Cirrus IV anterior ramus bearing only erect spines	**7**
2	Rostrum much longer than other parietes	**3**
–	Rostrum approximately as long as other parietes	**5**
3	Radii absent	***M. orcutti***
–	Radii present	**4**
4	Scutum with radius-like ledge on occludent margin	***M. koreanus***
–	Scutum occludent margin normal, lacking ledge	***M. declivis***
5	Parietes costate	***M. costatus***
–	Parietes not costate	**6**
6	Basal margin of all parietes rounded; tergal articular ridge low, articular groove open	***M. robinae***
–	Basal margin of laterals more or less straight; tergal articular ridge overhanging articular groove	***M. nebrias***
7	Radii broad, conspicuous; tergal spur narrow, longer than wide, cirrus IV pedicel without erect spines on anterodistal margins	***M. porphyrophilus* sp. nov.**
–	Radii absent or very narrow, tergal spur wider than long, cirrus IV pedicel with erect spines on anterodistal margins	**8**
8	Rostrum with median furrow, elongated basal portion tapering, very narrow	**10**
–	Rostrum without median furrow, basal portion wedge-shaped	***M. cuneiformis***
9	Basal membrane with spine-like processes	***M. brachialis***
–	Basal membrane without spine-like processes	***M. longirostrum***

## Supplementary Material

XML Treatment for Membranobalanus

XML Treatment for Membranobalanus porphyrophilus

## References

[B1] Atlas of Living Australia (2019) *Spheciospongia purpurea* (Lamarck, 1815) records. https://bie.ala.org.au/species/urn:lsid:biodiversity.org.au:afd.taxon:2a815069-da70-4531-9822-74e3ca64816d [Accessed on: 2019-7-22]

[B2] Barnard KH (1924) Contributions to the crustacean fauna of South Africa. No. 7, Cirripedia. Annals of the South African Museum 20: 1–103. https://biodiversitylibrary.org/page/11101069

[B3] Bergquist PR (1978) Sponges. Hutchinson & Co. Ltd, London, 267 pp.

[B4] Carter HJ (1885) Descriptions of sponges from the neighbourhood of Port Phillip Heads, South Australia. Annals and Magazine of Natural History (Series 5) 15(86): 107–117. 10.1080/00222938509459306

[B5] Chen H-N Høeg JT Chan BKK (2013) Morphometric and molecular identification of individual barnacle cyprids from wild plankton: an approach to detecting fouling and invasive barnacle species, Biofouling 29(2): 133–145. 10.1080/08927014.2012.753061 23327366

[B6] Cignoni P Callieri M Corsini M Dellepiane M Ganovell F Ranzuglia G (2008) Meshlab: an open-source mesh processing tool. In: Scarano V, De Chiara R, Erra U (Eds) Eurographics Italian Chapter Conference, Salerno (Italy), 2008, The Eurographics Association, 129–136. 10.2312/LocalChapterEvents/ItalChap/ItalianChapConf2008/129-136

[B7] Daniel A (1955) The Cirripedia of the Madras coast. Bulletin of the Madras Government Museum (New Series) Natural History Section 6: 1–40. http://www.e-books-chennaimuseum.tn.gov.in/chennaimuseum/images/248/book.html#p=2

[B8] Darwin C (1854) A monograph on the subclass Cirripedia with figures for all species. The Balanidae, the Verrucidae etc. The Ray Society, London, 681 pp http://darwin-online.org.uk/content/frameset?itemID=F339.2&viewtype=side&pageseq=1

[B9] D’Orbigny AD (1851) Cours élémentaire de paléontologie et de géologie stratigraphiques. Vol. 1(1), Masson, Paris, 382 pp https://gallica.bnf.fr/ark:/12148/bpt6k6260677z.texteImage

[B10] Grant RE (1826) Notice of a new zoophyte (*Cliona celata* Gr.) from the Firth of Forth. Edinburgh New Philosophical Journal 1: 78–81. https://www.biodiversitylibrary.org/page/2471191

[B11] Hiro F (1936) Report on the Cirripedia collected in Malayan waters by the Ship “Zuihomaru.” Japan Journal of Zoology 3: 621–636.

[B12] Hoek PPC (1913) The Cirripedia of the Siboga Expedition. B. CirripediaSessilia Siboga Expeditie Monographe XXXIb E.J. Brill, Leiden, 129–275. 10.5962/bhl.title.12959

[B13] Ilan M Loya Y Brickner I Kolbasov GA (1999) Sponge-inhabiting barnacles on Red Sea coral reefs. Marine Biology 133: 709–716. 10.1007/s002270050512

[B14] Innocenti G (2006) Collections of the Natural History Museum, Zoological Section “La Specola” of the University of Florence. XXIII. Crustacea, Class Maxillopoda, Subclass Thecostraca, Infraclass Cirripedia. Atti della Società Toscana di Scienze Naturali, Memorie serie B 113: 1–11. http://www.stsn.it/images/pdf/serB113/01.pdf

[B15] Jameson HL (1901) On the identity and distribution of the mother of pearl oysters with a revision of the subgenus Margaritifera. Proceedings of the Zoological Society of London 1901(1): 372–394. 10.1111/j.1469-7998.1901.tb08552.x

[B16] Jones DS (2000) CrustaceaCirripediaThoracica: Chionelasmatoidea and Pachylasmatoidea (Balanomorpha) of New Caledonia, Vanuatu and Wallis and Futuna islands, with a review of all currently assigned taxa. Mémoires du Muséum National d’Histoire Naturelle 184: 141–283.

[B17] Kim HS Kim IH (1983) A new species of *Membranobalanus*CrustaceaCirripedia from Korean waters. Korean Journal of Zoology 26: 41–47.

[B18] Kolbasov GA (1993) Revision of the genus *Acasta* Leach (Cirripedia: Balanoidea). Zoological Journal of the Linnean Society 109: 395–427. 10.1111/j.1096-3642.1993.tb00307.x

[B19] Kolosváry G (1941) Revisione della collezione de Balanidi del Museo Zoologico della R. Universita de Firenze. Monitore Zoologico Italiano 52: 183–195.

[B20] Lamarck JBPA de Monet Comte de (1815) Suites des polypiers empâtés. Mémoires du Muséum National d’Histoire Naturelle, Paris 1: 69–80, 162–168, 331–340.

[B21] Leach WE (1817) The Zoological Miscellany; Being Descriptions of New, or Interesting Animals. Vol. 3. E. Nodder and Son, Covent Garden and London, 151 pp. [ps 121–149]

[B22] Limaye A (2012) Drishti: a Volume Exploration and Presentation Tool. Proceedings of the Society of Photo-Optical Instrumental Engineers 8506, Developments in X-Ray Tomography VIII, 85060X. 10.1117/12.935640

[B23] Marshall W (1892) Spongiologische Beiträge. In: Festschrift zur siebzigsten Wiederkehr des Geburtstages von Rudolf Leuckart. C.F. Winter, Leipzig, 36 pp 10.5962/bhl.title.61018

[B24] Meyer C Geller J Paulay G (2005) Fine scale endemism on coral reefs: Archipelagic differentiation in turbinid gastropods. Evolution 59: 113–125. 10.1111/j.0014-3820.2005.tb00899.x 15792232

[B25] Nardo GD (1833) Auszug aus einem neuen system der spongiarien, wonach bereits die aufstellung in der Universitäts-Sammlung zu Padua gemacht ist. Isis, oder Encyclopädische Zeitung. Oken, Jena, 519–523.

[B26] Newman WA Ross A (1976) Revision of the balanomorph barnacles; including a catalogue of the species. San Diego Natural History Society Memoirs 9: 1–108. https://www.biodiversitylibrary.org/page/4328558

[B27] Pilsbry HA (1907) Notes on some Pacific cirripedes. Proceedings of the Academy of Natural Sciences of Philadelphia 59: 360–362. https://www.jstor.org/stable/4063151

[B28] Pilsbry HA (1916) The sessile barnacles (Cirripedia) contained in the collections of the U.S. National Museum; including a monograph of the American species. Bulletin of the United States National Museum, Washington: 93: 1–366. 10.5479/si.03629236.93.1

[B29] Prem-Kumar VK Daniel A (1968) A new species of operculate barnacle of the sub-genus *Membranobalanus* (Cirripedia, Thoracica) from sponges in the Indian seas. Crustaceana 14: 147–150. 10.1163/156854068X00520

[B30] Rosell NC (1972) Some barnacles (CirripediaThoracica) of Puerto Galera found in the vicinity of the U. P. Marine Biological Laboratory. Philippines University Natural and Applied Science Bulletin 24: 143–285.

[B31] Rosell NC (1973) On two less well-known balanids (Cirripedia, Thoracica) from the Sulu Archipelago, Philippines. University of the Phillippines Natural Science Research Center Technical Report 4: 1–12.

[B32] Rosell NC (1975) On two noteworthy balanids (CirripediaThoracica) from the Sulu Archipelago, Philippines. Crustaceana 29(2): 206–214. 10.1163/156854075X00216

[B33] Rützler K (1975) The role of burrowing sponges in bioerosion. Oecologia 19: 203–216. 10.1007/BF00345306 28309235

[B34] Schmidt O (1870) Grundzüge einer Spongien-Fauna des atlantischen Gebietes. Wilhelm Engelmann, Leipzig, 88 pp.

[B35] Semple TL Peakall R Tatarnic NJ (2019) A comprehensive and user‐friendly framework for 3D data visualisation in invertebrates and other organisms. Journal of Morphology 280(2): 223–231. 10.1002/jmor.20938 30653713 PMC6590182

[B36] Sulistiono S Kawaroe M Madduppa H Prabowo RE (2014) Karakteristik morfologi teritip spons Indonesia. Depik 3(2): 178–186. 10.13170/depik.3.2.1553

[B37] Suhaimi A (1966) A new species of *Balanus* (Crustacea: Cirripedia) from Singapore. Bulletin of the National Museum Singapore 33: 65–69.

[B38] Topsent E (1918) Éponges de San Thomé. Essai sur les genres *Spirastrella*, *Donatia* et *Chondrilla*. Archives de Zoologie Expérimentale et Générale 57: 535–618

[B39] Utinomi H (1968) Pelagic, shelf and shallow water cirripedia from the Indo West Pacific. Videnskabelige Meddelelser fra Dansk Naturhistorisk Forening 131: 161–186.

[B40] Van Syoc RJ (1988) Description of *Membranobalanus robinae*, a new species of sponge barnacle (Cirripedia, Archaeobalanidae) from Baja California, with a key to the genus. Proceedings of the Biological Society of Washington 101: 832–837. https://www.jstor.org/stable/20106147

[B41] Van Syoc RJ Van Soest RW Xavier JR Hooper JN (2015) A phylogenetic overview of sponge-inhabiting barnacles and their host specificity (Crustacea, Cirripedia). Proceedings of the California Academy of Sciences (Series 4) (62): 331–357.

[B42] Van Syoc RJ Winther R (1999) Sponge-inhabiting barnacles of the Americas: a new species of *Acasta* (Cirripedia, Archaeobalanidae), first record from the Eastern Pacific, including discussion of the evolution of cirral morphology. Crustaceana 72: 467–486. 10.1163/156854099503528

[B43] Vicente VP Rützler K Carballeira NM (1991) Comparative morphology, ecology, and fatty acid composition of West Indian *Spheciospongia* (Demospongea). Marine Ecology 12(3): 211–226. 10.1111/j.1439-0485.1991.tb00254.x

[B44] Vosmaer GCJ (1911) . The Porifera of the *Siboga* Expedition: 2. The genus *Spirastrella* Siboga Expeditie Monographe. E.J. Brill, Leiden, 1–69. https://biodiversitylibrary.org/page/2034921

[B45] Wibowo RA Prabowo RE Nuryanto A (2011) Biodiversitas teritip yang hidup pada spons di Perairan Pantai Kepulauan Karimunjawa. In: Prosiding Kongres dan Seminar Masyarakat Taksonomi Kelautan Indonesia, Jakarta 20–22 September 2011, 219–235.

[B46] Zullo VA Beach DB (1973) New species of *Membranobalanus* Hoek and *Hexacreusia* Zullo (Cirripedia, Balanidae) from the Galapagos Archipelago. Los Angeles County Natural History Museum Contributions in Science 249: 1–16. https://biodiversitylibrary.org/page/52110555

[B47] Zullo VA Standing JD (1983) Sponge-inhabiting barnacles (Cirripedia: Archaeobalanidae) of the Carolinian Province, southeastern United States, with the description of a new species of *Membranobalanus* Pilsbry. Proceedings of the Biological Society of Washington 96: 468–477. https://www.jstor.org/stable/20106147

